# Increased risk of malignancies in a population-based study of 818 soft-tissue sarcoma patients

**DOI:** 10.1038/sj.bjc.6603401

**Published:** 2006-09-26

**Authors:** J Fernebro, A Bladström, A Rydholm, P Gustafson, H Olsson, J Engellau, M Nilbert

**Affiliations:** 1 Department of Oncology, Institute of Clinical Sciences, Lund University Hospital, Lund, Sweden; 2 Department of Cancer Epidemiology, Institute of Clinical Sciences, Lund University Hospital, SE-221 85 Lund, Sweden; 3 Department of Orthopedics, Institute of Clinical Sciences, Lund University Hospital, SE-221 85 Lund, Sweden

**Keywords:** epidemiology, soft-tissue sarcoma, multiple primary malignancies, population-based, cancer risk

## Abstract

Soft-tissue sarcomas (STS) have been associated with various rare cancer syndromes and occur at increased frequencies in survivors of childhood cancer. Also adult patients with STS have been suggested to be at an increased risk of additional malignancies. After exclusion of syndrome-associated and radiation-induced sarcomas, we studied multiple primary malignancies in a population-based cohort of 818 patients with primary STS of the extremities and the trunk wall. In total, 203 other malignancies developed in 164 (20%) patients median 10 (0–32) years before and median 4 (0–35) years after the sarcoma diagnosis. Standardised morbidity ratios (SMRs) were determined for primary malignancies following a STS. Hereby individuals who had developed a STS were identified to be at increased risk of second primary malignancies (SMR for all malignant tumours=1.3; 95% CI=1.0–1.5; *P*=0.02) with STS being the only specific tumour type that occurred at an increased risk (SMR=17.6; 95% CI=8.1–33.5; *P*<0.001). Hence, this population-based series demonstrates a high frequency of second primary tumours among STS patients and indicates a particularly increased risk of developing a new STS.

Soft-tissue sarcomas (STS) represent less than 1% of all malignancies and constitute a group of biologically heterogeneous tumours. Some subtypes, for example, synovial sarcoma and myxoid liposarcoma are characterised by specific gene fusions, whereas others, for example, pleomorphic liposarcoma, leiomyosarcoma, and undifferentiated pleomorphic sarcoma/malignant fibrous histiocytoma (MFH) carry multiple complex genetic alterations (Helman and Meltzer, 2003). An increased risk of STS has been found in survivors of various types of childhood cancers, including retinoblastoma, leukaemia, Wilms' tumour, and Hodgkin's lymphoma (Breslow *et al*, 1995; Beaty *et al*, 1995; Moll *et al*, 1997; Neglia *et al*, 2001; Bisogno *et al*, 2004; Fletcher *et al*, 2004; Kleinerman *et al*, 2005). Also survivors of childhood STS, osteosarcoma, and Ewing sarcoma have been shown to be at increased risk of multiple primary malignancies, including bone sarcomas and STS (Heyn *et al*, 1993; Pratt *et al*, 1997; Neglia *et al*, 2001; Paulussen *et al*, 2001; Bacci *et al*, 2005; Cohen *et al*, 2005; Bassal *et al*, 2006). Development of multiple primary malignancies may also represent a side effect of chemotherapy or radiotherapy, which yield an increased risk of secondary malignancies, including STS (Brady *et al*, 1992; Kony *et al*, 1997; Neglia *et al*, 2001; Sheppard and Libshitz, 2001; Cormier and Pollock, 2004; Kirova *et al*, 2005; Thijssens *et al*, 2005; Virtanen *et al*, 2006). Children with STS who have been treated with radiotherapy have a nearly eight-fold risk of developing a second malignant neoplasm, including bone and STS (Cohen *et al*, 2005).

Based on current knowledge heredity, probably accounts for only a small subset of STS, although these tumours have been associated with several hereditary syndromes (Lynch *et al*, 2003). Among these, the most well-known are neurofibromatosis due to mutations in the *NF1* gene with an increased risk for neurofibrosarcomas and the Li–Fraumeni syndrome caused by germline mutations in *TP53* with increased risks for STS, leukaemia, brain tumours, and breast cancer (Lynch *et al*, 2003). However, STS have been associated also with premature ageing within the Werner syndrome and Rothmund–Thomson syndrome due to mutations in the genes *WRN* and *RECQ4* (Yu *et al*, 1996; Lindor *et al*, 2000) and has been described to develop in melanoma syndrome kindreds with *CDKN2A* mutations and in Lynch syndrome families with mutations in mismatch-repair genes (Sijmons *et al*, 2000; Lynch *et al*, 2002, 2003; Medina Arana *et al*, 2002; Hirata *et al*, 2006). In addition, STS occur in familial forms with yet unidentified modes of inheritance and underlying genetic causes.

Two recent studies have described development of multiple primary malignancies in adult STS patients, and also suggested that STS patients may be at risk of developing a second primary STS (Merimsky *et al*, 2001; Tateishi *et al*, 2005). We used the southern part of the Swedish Cancer Registry to identify all individuals with STS during the time period 1964–2001 to characterise development of multiple primary malignancies in this cohort and to assess the risk of a second primary malignancy after STS.

## MATERIALS AND METHODS

### The STS cohort

The National Swedish Cancer Registry is based on the International Classification of Diseases (ICD) codes and is, because of mandatory cancer registration by both pathologists and clinicians, population-based and estimated to contain 99% of all malignant diagnoses. The study is based on the southern Sweden (currently 1.7 million inhabitants) part of the registry. All individuals diagnosed with primary STS of the extremities and trunk wall during 1964–2001 were identified. Medical records were in all patients scrutinised for pertinent data, that is, syndrome-associated diseases, and previous treatment with radiotherapy and/or chemotherapy, and all tumours have repeatedly been reviewed by sarcoma pathologists at our tumour centre, and the vast majority of these tumours had been reviewed by the Scandinavian Sarcoma Review Board (Rydholm, 1983; Gustafson, 1994; Engellau, 2004). We excluded children (<16 years of age at diagnosis), sarcomas associated with neurofibromatosis type I, dermal sarcomas, and STS that developed in previously irradiated fields or were associated with Stewart–Treves syndrome. Hereafter, 824 patients with primary STS of the extremities or the trunk wall remained, but six patients who died at the time of STS diagnosis were excluded, and the study was thus based on 818 individuals (Table 1). The histopathological reports were reviewed to confirm primary tumour origin, location, and subtype.

### Development of other primary malignancies

All other malignancies that had developed in these 818 patients during 1958–2004 were identified in the Cancer Registry. The histopathological reports regarding the other malignancies were collected from 169 cases, whereas data in 34 cases relied on information from the Cancer Registry only, most often because of missing data at the local pathology departments. All previous radiotherapy fields were re-evaluated regarding overlap with the site of the secondary tumour, but only one tumour – a squamous cell carcinoma that developed 26 years after irradiation for a liposarcoma of the groin – was excluded since it was regarded as radiation-induced. Soft-tissue sarcoma that developed at another anatomical site before the development of any metastases (in the lungs or at other sites such as lymph nodes or skeletal metastases) were regarded as multiple primary STS, whereas STS that developed at the same location as the primary tumour or in patients with known metastases were regarded as local recurrences and were thus not included in the analysis (Antonescu *et al*, 2000). Furthermore, among the patients with second primary STS, five of seven remained free of metastases after long-term follow-up.

### Risk assessment

The patients within the cohort were followed from the time of STS diagnosis to death (when they were censored) or until 31st December 2004 when the study was closed. The cancer incidence within the southern Swedish part of the Cancer Registry was used as a reference. The analysis was stratified by sex, calendar year, and 5-year age groups, that is, the cancer incidence in the STS cohort was compared to the cancer incidence in the reference population. The risk estimates were based only on tumours that developed after the STS because of difficulties in compensating for death rates from prior malignancies and after exclusion of a squamous cell carcinoma of the skin that developed within a previously irradiated field. Standardised morbidity ratios (SMRs) were calculated by dividing the observed number of cancer cases by the expected number for the total cohort (818 patients) and also for subgroups with respect to sex, age at STS diagnosis, and with exclusion of the 77 individuals with another primary malignancy before the STS diagnosis. Ethical permission for the study was granted from the Lund University ethics committee.

## RESULTS

### Primary tumours preceding or following the STS

Among the 818 patients, 164 (20%) developed 203 additional primary malignancies preceding or following the STS (Table 1). The median age at the STS diagnosis was 66 (range 16–98) years and the median age at the first malignant diagnosis among patients with multiple tumours, irrespective of tumour type, was 69 (31–91) years. The other malignancies developed median 10 (0–32) years before and median 4 (0–35) years after the sarcoma diagnosis. One additional malignancy was found in 131 individuals, two additional malignancies in 27, and three additional malignancies developed in six patients. In relation to the type of STS, multiple primary malignancies occurred in 93 of 306 (30%) patients with MFH, in 31 of 143 (22%) patients with leiomyosarcomas, 19 of 81 (23%) with liposarcomas, six of 58 (10%) with synovial sarcomas, 19 of 35 (54%) with malignant peripheral nerve sheath tumours (MPNST), and in 35 of 161 (22%) patients with STS of other subtypes.

Primary malignancies (*n*=90) that developed prior to the STS diagnosis were identified in 77 patients, nine of whom had developed two and two of whom had developed three primary malignancies. The most common cancer types were breast cancer (*n*=19), prostate cancer (*n*=10), and malignant melanoma (*n*=10) (Table 2). Second primary malignancies (*n*=113) after the STS were identified in 96 patients. Of these, 82 patients developed one additional malignancy, 11 developed two, and three patients developed three additional malignancies. The second primary malignancies developed at median age 76 (45–95) years (Table 1). The most frequent tumour types after the STS diagnosis were prostate cancer (*n*=18) and colorectal cancer (*n*=16) (Table 2). A second STS at another anatomical location developed in seven patients mean 4 (range 2–5) years after the primary diagnosis, and two of these patients developed a third subsequent STS (Table 3). Of these nine tumours, three were of another histopathologic subtype than the primary tumour. These patients did not have metastases at other sites and only two of seven individuals developed subsequent lung metastases after 47 and 50 moths, respectively (Table 3). Among the 96 patients who developed other malignancies following the STS, radiotherapy had been administered preoperatively to one patient and postoperatively to 22 patients, and chemotherapy had been given preoperatively to one patient and postoperatively to three patients.

### SMR calculations

The follow-up for the whole cohort encompassed 6910 person years and during this time 113 malignancies developed compared to the expected number of 90, which corresponds to a SMR for all malignancies of 1.3 (95% CI=1.0–1.5; *P*=0.02) (Table 2). The only specific tumour type that developed at increased risk was STS with a SMR of 17.6 (95% CI=8.1–33.5; *P*<0.001) (Table 2). When the 77 individuals with another malignancy before the STS were excluded, similar results were obtained with a significantly increased risk of all malignancies (SMR, all tumours=1.3; 95% CI=1.0–1.5; *P*=0.03) and a specifically increased risk of STS (SMR=17.1; 95% CI=7.4–33.7). The increased risk of second primary tumours was particularly high in individuals who developed a STS after age 64 (SMR, all tumours=1.4; 95% CI=1.2–1.8; *P*<0.01) and an increased risk of lung cancer was identified in this subgroup (SMR=2.2; 95% CI=0.9–4.5; *P*=0.04).

## DISCUSSION

Several causes, including treatment-related factors and heredity, are likely to underlie development of multiple primary malignancies (Kony *et al*, 1997). Based on data from the National Swedish Cancer Registry, two or more primary malignancies develop in 8–10% of all cancer patients, whereas three or more malignancies are found in less than 1% of the patients (Swedish board of health and welfare; http://www.sos.se/sosmenye.htm). Centre-based studies have suggested that 7–10% of adult STS patients develop multiple primary malignancies (before or after the sarcoma diagnosis) (Hartley *et al*, 1993; Merimsky *et al*, 2001; Tateishi *et al*, 2005). Development of a third primary tumour has in the previous studies been reported in 1.6–2% of the patients (Merimsky *et al*, 2001; Tateishi *et al*, 2005). In this study, one second primary malignancy developed in 16% of the patients and two or more other malignancies were diagnosed in 4% of the patients, hence at higher rates than among cancer patients in general as well as in previous reports on adult STS patients. Since we excluded individuals with hereditary syndromes and STS within irradiated fields, and since chemotherapy had only been administered to four patients, genetic and treatment-related factors are not likely to explain the occurrence of multiple primary tumours in our series.

The frequency of multiple primary malignancies varied between the different STS subtypes, but may be influenced by survival differences for the different histopathological subtypes. The lowest rate of second malignancies was observed in patients with synovial sarcomas (only six of 58 developed another malignancy), which is in line with the results from Tateishi *et al* (2005), who did not observe any second malignancies in individuals with synovial sarcoma. Two previous studies have suggested that the risk of development of multiple primary malignancies is particularly high among STS patients with MFHs, including myxofibrosarcoma (Merimsky *et al*, 2001; Tateishi *et al*, 2005). In our series, multiple primary tumours most frequently occurred in patients diagnosed with MPNST (19 of 35) followed by MFH (93 of 306) (Table 1). Since patients with neurofibromatosis type I were excluded from the study, the high frequency of multiple primary malignancies in patients with MPNST is not likely to be explained by this syndrome. Patients with neurofibromatosis type I are at a higher risk of several tumour types, including MPNST, chronic myelogenous leukaemia and gliomas (Listernick *et al*, 1999; Korf, 2000). However, none of these tumour types were observed among patients diagnosed with MPNST, in whom the most common second tumour types were breast cancer followed by prostate cancer, STS, and tumours of the urinary tract. Merimsky *et al* (2001) identified renal cell cancer and STS as the most common second tumour types in their study of a mixed STS cohort, and although we confirm an increased frequency of STS, we did not find any increased risk of renal cell cancer. Tateishi *et al* (2005) showed that the risk of multiple malignancies increased with the age at diagnosis. This finding seems to be in accordance with our study, since patients diagnosed with a STS after age 64 indicated a larger increase compared to individuals below 65 years of age.

We analyzed the risk of multiple primary tumours in patients previously diagnosed with STS. Overall, 113 second primary malignancies following the sarcoma developed in 96 (12%) patients. An increased SMR (1.3) for all malignant tumours was found, with the only specific tumour type to develop at an increased SMR (17.6) being a second STS. Among the seven patients who developed a second STS, three of nine tumours were of another histopathologic subtype and two individuals developed three STS (Table 3). Although we required that the second STS should be at another anatomical location and should develop before any lung metastases, it is difficult to determine whether multiple STS in the same individual represent multiple primary tumours or rather an unusual pattern of metastatic disease. Indeed, occasional cases with soft-tissue metastases from liposarcomas, sometimes several years after the primary tumour, have been reported (Antonescu *et al*, 2000), but only one of the patients in our series presented with multiple liposarcomas. The observation of STS at different anatomical sites and with different histopathology is in line with reports on multiple primary STS in a small subset (0.2%) of STS patients (Antonescu *et al*, 2000; Grobmyer *et al*, 2004).

Because of the rarity of STS, specific associations with other cancer types may be difficult to recognise. In the context of familial sarcoma, an association with several tumour types has been described and besides the well-known syndromes such as neurofibromatosis and Li–Fraumeni syndrome, STS have been observed as a rare tumour manifestation in various other hereditary syndromes together with, for example, melanoma, pancreatic cancer, and colorectal cancer (Lynch *et al*, 2003). Although no specific tumour type other than STS occurred at significantly increased risk in the present study, malignant melanoma developed in 12 patients and pancreatic cancer in four. Development of STS has been described in association with the *CDKN2A* mutation associated with the familial atypical multiple-mole melanoma syndrome (Lynch *et al*, 2002, 2003). Also, endometrial cancer occurred in 11 women and colorectal cancer in 21 individuals, and some of these cases could signify Lynch syndrome, since STS has been described herein (Sijmons *et al*, 2000; Medina Arana *et al*, 2002; Hirata *et al*, 2006). Data on family history of cancer are not available in this material, but because of the links of STS described to various cancer syndromes, we suggest that a family history of cancer should be obtained in STS patients. Specific attention should perhaps be given to cases of melanoma, endometrial cancer, and colorectal cancer in the individual or in the family, since these tumour types may be prevented through screening programmes, whereas the occurrence of multiple primary STS needs further characterisation in order to clarify its causes.

## Figures and Tables

**Table 1:** Clinical data in whole cohort and for the 203 other malignancies in 164 patients

		**Second primary malignancies**	
	**Whole cohort (*n*=818)**	**Before sarcoma diagnosis (*n*=90)**	**After sarcoma diagnosis (*n*=113)**
Sex (male : female)	440 : 378	36 : 41	55 : 41
Median age at sarcoma diagnosis (range)	66 (16–98)	74 (51–94)	69 (32–91)
Median age at diagnosis of other tumour (range)		64 (31–91)	76 (45–95)
			
*Tumour location* a			
Lower extremity	519	59	68
Upper extremity	176	25	36
Trunk wall	95	6	9
			
*Histotypes no.*	(% of whole cohort)	(% of subtype)	(% of subtype)
MFH	306 (37)	43 (14)	50 (16)
Leiomyosarcoma	143 (17)	11 (8)	20 (14)
Synovial sarcoma	58 (7)	1 (2)	5 (10)
Liposarcoma	81 (10)	5 (6)	14 (17)
MPNST	35 (4)	8 (23)	11 (31)
Mal haemangiopericytoma	35 (4)	3 (9)	6 (17)
Other^b^	160 (20)	19 (12)	7 (4)

a Data missing in 28 cases.

b Includes extraskeletal osteosarcoma, extraskeletal Ewing sarcoma, extraskeletal myxoid chondrosarcoma, soft-tissue sarcoma UNS, epitheloid sarcoma, alveolar soft part sarcoma, angiosarcoma, clear-cell sarcoma, fibrosarcoma, haemangiosarcoma, and rhabdomyosarcoma.

**Table 2:** Second primary malignancies preceding or following the soft-tissue sarcoma diagnosis

**Tumour type**	**Number observed before STS**	**Number observed after STS**	**Number expected after STS**	**SMR**	**95% CI**
All malignant tumours^a^	90	113	90.2	**1.3** *	**1.0**–**1.5**
Prostate cancer	10	18	15.3	1.2	0.7–1.9
Colorectal cancer	5	16	12.6	1.3	0.7–2.1
Urinary tract	6	9	5.5	1.7	0.8–3.1
Lung cancer	1	9	6.4	1.4	0.7–2.7
Soft tissue	0	9	0.5	**17.6** **	**8.1**–**33.5**
Breast cancer	19	8	8.6	0.9	0.4–1.8
Endometrial cancer	6	5	1.8	2.8	0.9–6.4
Gastric cancer	1	5	3.6	1.4	0.5–3.2
Non-Hodgkin's lymphoma	5	4	2.7	1.5	0.4–3.8
Skin cancer	8	4	6.7	0.6	0.2–1.5
Pancreatic cancer	0	4	2.2	1.8	0.5–4.6
Renal cell cancer	1	3	2.2	1.4	0.3–4.0
Malignant melanoma	10	2	2.5	0.8	0.1–2.9
Hepatobiliary cancer	0	3	2.0	1.5	0.3–4.3
Chronic lymphocytic leukaemia	2	2	0.9	2.3	0.3–8.3
Brain tumour	0	1	1.9	0.5	0.01–2.9
Thyroid gland	3	0	0.4	0.0	0.0–8.6
Ovarian cancer	3	1	1.5	0.7	0.02–3.8

CI=confidence interval; SMR=standardised morbidity ratio; STS=soft-tissue sarcoma.

a In addition to the tumours listed below, 10 additional malignancies, for example, head and neck cancer, developed.

* *P*=0.02.

** *P*<0.001.

**Table 3:** Patients with multiple primary soft-tissue sarcomas

**Case no.**	**Sex**	**First STS**	**Site**	**Depth**	**Age (years)**	**Second STS**	**Site**	**Depth**	**Age**	**Third STS**	**Site**	**Depth**	**Age (years)**	**Pulm. met. (months)^a^**
1	F	MFH	Upper arm R	D	82	MFH	Buttock L	D	84	MFH	Scalp	S	85	47
2	F	MFH	Shoulder R	D	76	MFH	Thigh L	D	81	LMS	Lower arm R	D	85	None
3	F	MFH	Upper arm L	S	74	MFH	Lower leg R	S	79					None
4	M	MPNST	Trunk wall	S	46	AS	Upper arm	S	50					None
5	F	MPNST	Lower arm R	D	59	LMS	Thigh L	S	63					None
6	M	LS	Knee L	S	60	LS	Axilla R	D	62					None
7	M	LMS	Lateral thigh L	S	83	LMS	Medial thigh L	D	87					50

AS=angiosarcoma; D=deep; L=left; LMS=leiomyosarcoma; LS=liposarcoma; MFH=malignant fibrous histiocytoma; MPNST=malignant peripheral nerve sheath tumour; R=right; S=superficial; STS=soft-tissue sarcomas.

a Time from first STS to diagnosis of pulmonary metastases.
